# Development and validation of a multimodal clinical-radiomics-deep learning nomogram based on automated chest CT segmentation for classifying COPD severity: a multicenter study

**DOI:** 10.3389/fmed.2026.1831103

**Published:** 2026-05-13

**Authors:** Qiang Fei, Xiaoli Mei, Jiacheng Zhao, Yili Xing, Yuanbin Li, Mei Yang, Jingnan Xue, Luying Qi, Yifeng Zheng, Hongxing Zhao, Hupo Bian

**Affiliations:** 1 Department of Radiology, The Linghu People's Hospital, Huzhou, Zhejiang, China; 2 Pulmonary Function Laboratory, The Linghu People's Hospital, Huzhou, Zhejiang, China; 3 Department of Information System Engineering, Tokyo Denki University, Tokyo, Japan; 4 Department of Nephrology, The Linghu People's Hospital, Huzhou, Zhejiang, China; 5 Department of Radiology, The First Affiliated Hospital of Huzhou University, Huzhou, Zhejiang, China; 6 School of Medicine (School of Nursing), Huzhou University, Huzhou, Zhejiang, China; 7 Department of Radiology, Huzhou Central Hospital Affiliated to Huzhou University, Huzhou, Zhejiang, China; 8 Huzhou Key Laboratory of Precise Diagnosis and Treatment of Urinary Tumors, Huzhou, Zhejiang, China; 9 Department of Radiology, Nanhu District People’s Hospital, Jiaxing, Zhejiang, China

**Keywords:** chronic obstructive pulmonary disease, deep learning, nomogram, prediction model, radiomics

## Abstract

**Background and objective:**

Chronic obstructive pulmonary disease (COPD) is a widespread and severely disabling respiratory disorder that places a substantial burden on global healthcare systems. Precise determination of COPD severity staging is essential for effective patient management and treatment planning. This study seeks to develop and validate a comprehensive nomogram that combines clinical characteristics, whole-lung computed tomography (CT) radiomic features, and deep learning-derived features to classify COPD severity.

**Method:**

A retrospective analysis included 1,794 patients from three hospitals, spanning January 1, 2021, to May 30, 2025. Following fully automated segmentation of the entire lungs, radiomic features and three-dimensional deep learning features were extracted. A comprehensive nomogram was developed and validated, integrating radiomics features, deep learning features, and independent clinical factors. Model performance was assessed and compared using receiver operating characteristic (ROC) curves, the area under the ROC curve (AUC), decision curve analysis (DCA), and the DeLong test.

**Result:**

In the training, internal validation, and external validation cohorts, the area under the receiver operating characteristic curve (AUC) values for the clinical model were 0.634, 0.630, and 0.616, respectively; for the radiomics (Rad) model, 0.760, 0.729, and 0.704, respectively; for the deep learning (DL) model, 0.805, 0.757, and 0.752, respectively; for the radiomics-deep learning combined (DLR) model, 0.822, 0.740, and 0.759, respectively; and for the combined model, 0.839, 0.759, and 0.767, respectively. The combined model outperformed the individual clinical, radiomics, and three-dimensional deep learning models.

**Conclusion:**

This study constructed and validated a novel combined logistic regression model for identifying the severity of COPD by integrating the clinical characteristics of independent risk factors, the full lung radiomics features, and the deep learning features. It demonstrated the additional value of chest CT in evaluating lung structure and lung function status.

## Introduction

Chronic obstructive pulmonary disease (COPD) is a prevalent, preventable, and treatable condition marked by persistent respiratory symptoms and airflow limitation. It has emerged as the third leading cause of death worldwide, imposing a substantial burden on health systems and society (1–3). Research indicates that increased COPD severity correlates with a higher frequency of exacerbations, leading to elevated mortality rates (4). Furthermore, COPD frequently coexists with other chronic conditions, such as cardiovascular disease, osteoporosis, diabetes, lung cancer, and cachexia (5–8). These comorbidities collectively diminish patients’ quality of life and increase mortality. Treatment strategies for COPD vary according to disease severity (9), highlighting the importance of accurately assessing COPD severity to improve patient prognosis and reduce mortality.

Pulmonary function testing (PFT) remains the gold standard for diagnosing and staging COPD, categorizing severity into four stages: mild, moderate, severe, and very severe (5). Despite its status, PFT utilization remains low; in China, only 6.7% of individuals aged 40 and above have undergone PFT (10). Moreover, PFT has notable limitations: it reflects only a single dimension of airflow limitation and fails to capture regional lung tissue changes, such as emphysema, small airway disease, and airway remodeling (11). Meanwhile, detection tools such as the Lung Function Questionnaire (LFQ), the COPD Diagnosis Questionnaire (CDQ), and the COPD Population Screening Tool (COPD-PS) have demonstrated their practicality in identifying individuals at high risk of COPD. However, these tools mainly rely on self-reported symptoms and risk factors, which may lead to recall bias, and they cannot directly assess potential structural changes in the lungs. With the widespread adoption of lung cancer screening programs, chest computed tomography (CT) has become increasingly accessible, offering detailed anatomical information. Studies have shown that COPD of varying severities exhibits distinct CT morphological features. Quantitative imaging has been employed to assess COPD severity and predict coronary heart disease risk (12). Cho et al. (13) demonstrated that quantitative pulmonary vascular characteristics correlate with the severity and extent of emphysema in COPD. Leveraging CT images for COPD staging could thus enable timely “one-stop” medical interventions, yielding greater benefits (14). However, traditional CT assessment relies heavily on radiologists’ subjective visual evaluation of manifestations such as emphysema and airway wall thickening, making it challenging to detect complex image texture patterns imperceptible to the human eye.

In recent years, artificial intelligence has been increasingly applied in COPD research. Radiomics techniques quantify the heterogeneity, texture, and morphological features of lesion regions by extracting numerous quantitative features from medical images (15). Given COPD’s diffuse and heterogeneous nature, the entire lung is designated as the region of interest. Meanwhile, deep learning (DL) technology has made significant advances in medical image analysis. DL models automatically learn hierarchical deep feature representations from raw images, overcoming the limitations of manual feature engineering and excelling in image classification, segmentation, and prediction tasks (16). Combining deep learning with radiomics offers complementary advantages: deep learning mines deep implicit features automatically, while radiomics provides interpretable quantitative features, jointly enhancing predictive model performance.

Our team has found that a fusion model integrating radiomics and deep learning via one-stop chest CT can effectively predict the risk of concurrent coronary heart disease in COPD patients (17). We hypothesize that a fusion model combining whole-lung radiomics from chest CT with deep learning can identify COPD severity in routine clinical practice. This approach would enhance the value of chest CT, particularly for patients unable to undergo PFT, by providing both morphological information and pulmonary function assessment. To facilitate clinical use, we integrate automatic deep learning features with interpretable radiomics features to comprehensively explore effective information in CT images and combine it with key clinical factors to construct a nomogram model. This provides clinicians with a visual and quantitative individualized assessment tool.

## Methods and materials

### Patients and clinical data

This study has been approved by the Institutional Review Board of The First Affiliated Hospital of Huzhou University (Approval Number: 2025KYLL002-01). Given the retrospective nature of the study, the requirement for informed consent was waived. This study was conducted in accordance with the ethical principles outlined in the Declaration of Helsinki. Clinical data and tomographic images of eligible patients were collected from three hospitals from January 1, 2021, to May 30, 2025. During this period, a total of 1,794 patients diagnosed with chronic obstructive pulmonary disease (COPD) through pulmonary function tests (PFTs) at these three centers were included in this study.

The inclusion criteria were as follows: (1) Diagnosed with chronic obstructive pulmonary disease confirmed by pulmonary function tests; (2) Completion of pulmonary function tests and chest CT scans within 2 weeks; (3) Possession of complete thin-slice (1 mm) chest CT images. The exclusion criteria were as follows: (1) Incomplete clinical data, concurrent other thoracic diseases (such as pneumonia, atelectasis, pulmonary nodules or masses larger than 6 mm, and pleural effusion); (2) Concurrent any malignancy; (3) Presence of spinal implants or significant image artifacts, with poor image quality affecting diagnosis; (4) Lack of thin-slice chest CT images. We randomly assigned 1,313 patients from the first center to the training cohort (*n* = 919) or the internal validation cohort (*n* = 394) in a ratio of 7:3, and allocated 434 patients from the second center and 47 patients from the third center to the external validation cohort (*n* = 481). The external validation cohorts (Centers 2 and 3) were derived from two completely independent medical centers. Their CT acquisition equipment, scanning protocols, and patient populations were all different from those of Center 1, ensuring a rigorous assessment of the model’s generalization ability. Clinical information included age, body mass index (BMI), gender, smoking status, as well as laboratory test indicators such as C-reactive protein (CRP), erythrocyte sedimentation rate (ESR), and procalcitonin (PCT). For some missing values, we used the mean to fill them in, and for data with missing values accounting for more than 30% of the information, we did not include them in the final study.

### CT image acquisition and pulmonary function examination

Participants underwent non-enhanced CT scans using equipment from manufacturers such as Aquilion ONE TSX-301C and Brilliance CT 16. Axial CT images of the entire thorax were acquired during full inspiration (scanning parameters are detailed in Supplementary Data 1). Pulmonary function testing was conducted using the Ganshorn PowerCube. The diagnosis and classification of chronic obstructive pulmonary disease (COPD) adhered to the criteria outlined by the Global Initiative for Chronic Obstructive Lung Disease (GOLD) (18). COPD was defined as a post-bronchodilator ratio of forced expiratory volume in the first second (FEV1) to forced vital capacity (FVC) below 0.7. In this study, COPD severity was categorized into two stages according to GOLD criteria: mild to moderate airflow limitation (GOLD stages 1–2, FEV1 ≥ 50% of predicted value) and severe to very severe airflow limitation (GOLD stages 3–4, FEV1 < 50% of predicted value).

### Whole lung automatic segmentation

A deep learning model^1^ was employed to segment the left and right lungs, and the extracted regions were merged into a single region of interest (ROI). The specific algorithm process is detailed in Supplementary Data 2. To assess the consistency between automatic and manual segmentation results, 100 samples were randomly selected and independently evaluated by two chest radiologists, each with over 10 years of experience, using ITK-SNAP (version 3.8.0) (34).^2^ The Dice index was then calculated to objectively quantify the spatial overlap of contours and determine the agreement between fully automatic and manual segmentation results. Finally, automatic segmentation was applied to the remaining samples.

### Extraction and selection of radiomics features

We employed Pyradiomics^3^ to extract radiomic features from segmented lung regions. Before feature extraction, the images underwent a three-step preprocessing procedure to standardize them. First, the images were resampled to a resolution of 1 mm × 1 mm × 1 mm. Second, the gray-level values were adjusted to a 25 Gy level to mitigate the effects of varying slice thicknesses and reduce noise interference. Subsequently, features were extracted using Z-score normalization, yielding three categories of features: first-order, shape, and texture features. Statistical significance was evaluated using the t-test, and only features with a *p*-value less than 0.05 were retained. To address collinearity, we assessed the correlation between features using the Pearson correlation coefficient and excluded features with a correlation coefficient exceeding 0.9. Furthermore, within a 10-fold cross-validation framework, we used Lasso regression to optimize the feature set by determining the optimal regularization parameter *λ* and selecting features with non-zero coefficients. Finally, the radiomic score (Rad Score, RS) was computed as a linear combination of the retained features and their corresponding coefficients.

### Extraction and selection of deep learning features

In this study, the ResNet50 architecture was employed as a convolutional neural network (CNN) to extract deep learning features. Image intensity distributions were standardized using Z-score normalization, and the standardized images were used as inputs to the deep learning model. During training, real-time data augmentation techniques—such as random cropping and flipping—were applied to improve model robustness. For test images, preprocessing was restricted to normalization to ensure consistency during evaluation. A pre-trained CNN model was used to extract deep transfer learning (DTL) features from the largest region of interest (ROI) in each image, specifically from the penultimate layer. In the proposed model, the output probabilities computed by the CNN were defined as deep learning feature signatures. Given the complexity of the CNN, principal component analysis (PCA) was applied to reduce these features to a 512-dimensional space for improved manageability.

### Model construction and evaluation

**Figure 1:**
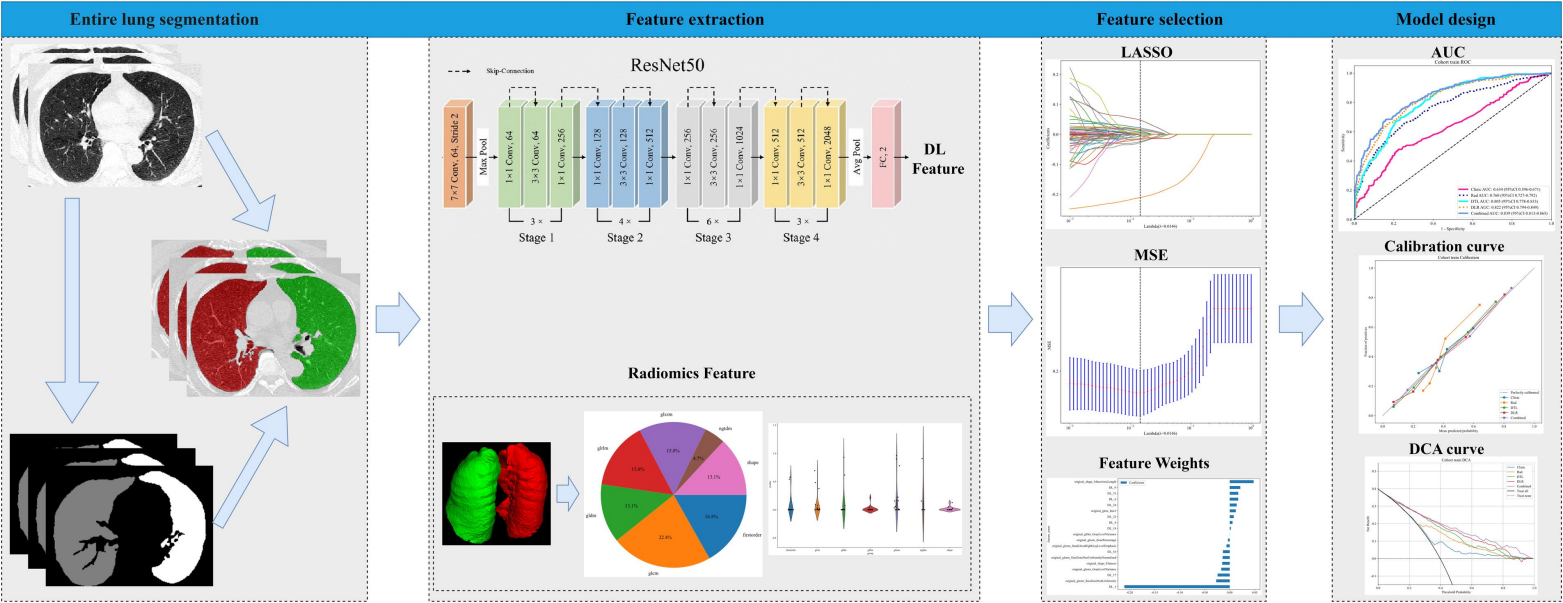
The flowchart of the entire research design.

Following feature selection, five distinct models were constructed. Logistic regression analysis was initially applied to clinical features to identify statistically significant predictors, which were then used to develop a clinical model. Using the selected radiomic and deep learning features, an imaging biomarker (Rad) model and a deep learning (DTL) model were constructed separately. To create a deep learning imaging biomarker (DLR) model, a fusion algorithm was employed to integrate deep learning features with radiomic features. To improve clinical applicability, univariate and stepwise multivariate analyses were performed on all clinical features to identify significant predictors. These selected clinical features were subsequently combined with the predictive results of the DLR model to develop a logistic regression (LR)-based linear model, termed the comprehensive model, which was effectively visualized through a nomogram. The models were evaluated using the area under the curve (AUC), accuracy, sensitivity, specificity, recall, and F1 score. Model performance was further assessed using positive predictive value (PPV) and negative predictive value (NPV). The AUCs of different models were compared using the DeLong test, and calibration was analyzed using calibration curves (Hosmer-Lemeshow test) to verify reliability. Decision curve analysis (DCA) was also conducted to evaluate the clinical utility of the predictive models, providing insights into their potential benefits in clinical settings. Figure 1 presents a flowchart of the entire study design.

### Statistical analysis

The Shapiro–Wilk test was employed to evaluate the normality of clinical characteristics. Continuous variables were analyzed using the t-test or the Mann–Whitney U test, depending on their distribution. Categorical variables were assessed using Chi-square (χ^2^) tests.

All data analyses were performed using Python 3.7.12. Statistical analyses utilized Statsmodels version 0.13.2, while PyRadiomics version 3.0.1 was used for radiomics feature extraction. Scikit-learn version 1.0.2 facilitated machine learning tasks, and PyTorch version 1.11.0 was employed for deep learning framework development, with performance optimized using CUDA version 11.3.1 and cuDNN version 8.2.1.

## Results

### Baseline characteristics and clinical feature screening of the patients

**Figure 2:**
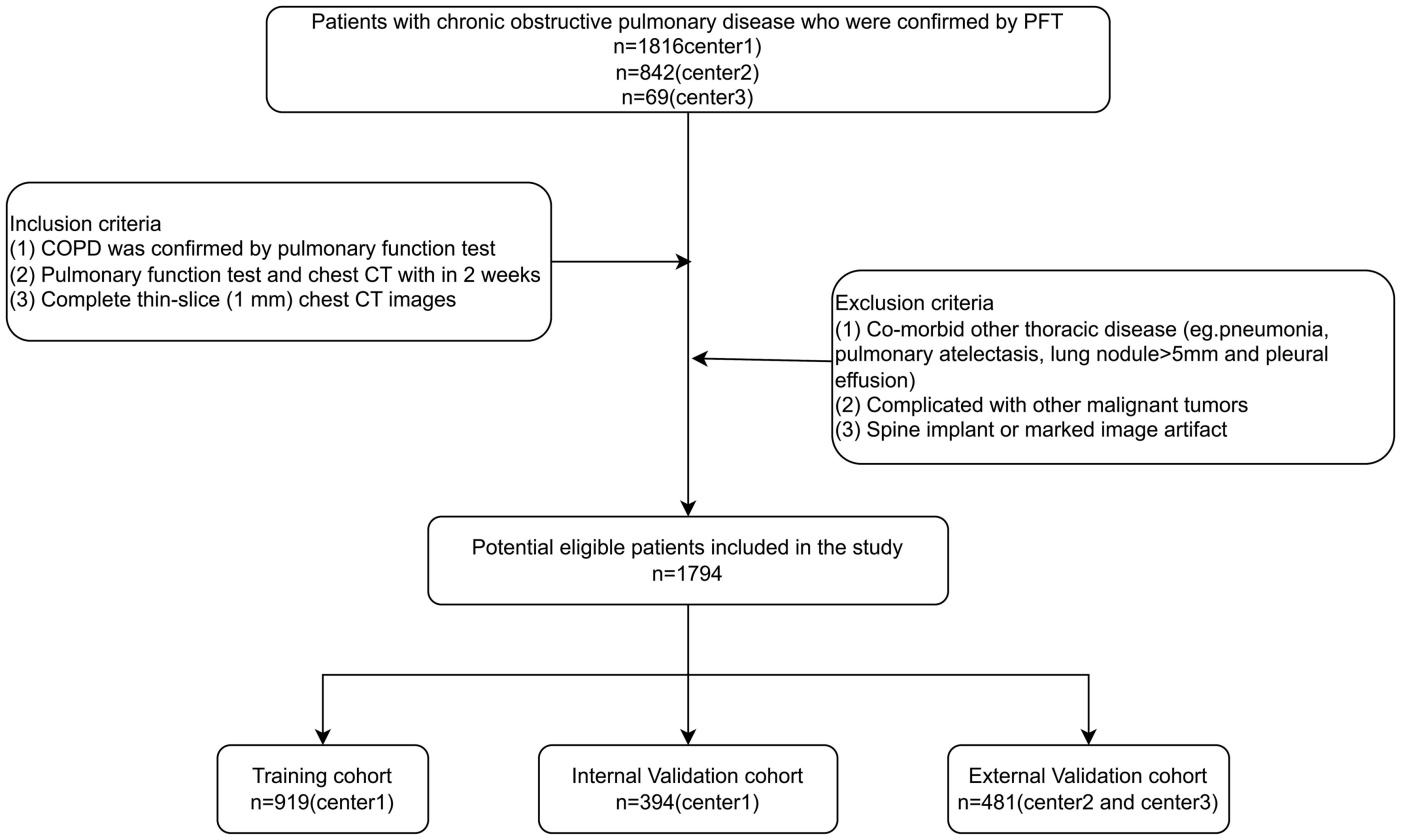
The flowchart of the patient’s selection.

Figure 2 presents the flowchart illustrating patient selection. As of May 30, 2025, a total of 2,658 patients diagnosed with COPD were included in the study. Following screening based on inclusion and exclusion criteria, the final cohort comprised 1,794 patients, including 276 females and 1,518 males. The mean age of the cohort was 74.02 years. The number of patients from the three hospitals was 1,313, 434, and 47, respectively. Among these patients, 989 had COPD classified as GOLD I–II, and 805 had COPD classified as GOLD III–IV. Table 1 provides details on the baseline clinical characteristics of the study cohort (standardized units for all indicators in Table 1 are available in Supplementary Data 5).

**Table 1:** Baseline characteristics of the study population.

Clinical factors	Training cohort (*n* = 919)			Internal validation cohort (*n* = 394)			External vaidation cohort (*n* = 481)		
	GOLD I–II (*N* = 554)	GOLD III–IV (*N* = 365)	*p* value	GOLD I–II (*N* = 228)	GOLD III–IV (*N* = 166)	*p* value	GOLD I–II (*N* = 207)	GOLD III–IV (*N* = 274)	*p* value
Age	74.38 ± 8.15	73.76 ± 8.11	0.387	75.04 ± 8.11	73.84 ± 8.35	0.127	73.33 ± 8.02	73.35 ± 7.49	0.96
BMI	508.90 ± 246.13	472.64 ± 256.58	0.045	545.96 ± 228.39	431.58 ± 239.93	<0.001	495.43 ± 230.98	473.70 ± 276.15	0.315
Albumin	39.66 ± 14.07	38.59 ± 4.08	0.012	39.13 ± 4.16	38.66 ± 4.26	0.275	38.05 ± 5.06	37.45 ± 4.32	0.19
Globulin	104.55 ± 42.37	98.36 ± 43.21	0.011	104.65 ± 46.47	94.98 ± 41.30	0.063	100.43 ± 39.72	90.53 ± 40.16	0.01
Triglyceride	1.16 ± 0.69	1.04 ± 0.69	<0.001	1.16 ± 0.69	1.41 ± 5.16	0.016	2.01 ± 12.97	1.24 ± 4.52	<0.001
Alkaline Phosphatase	89.31 ± 67.48	83.30 ± 41.92	0.056	87.33 ± 48.09	87.18 ± 37.23	0.801	80.39 ± 59.90	76.89 ± 27.77	0.761
White blood cell count	6.74 ± 3.63	7.31 ± 3.62	0.018	6.48 ± 2.35	6.95 ± 3.18	0.469	6.68 ± 3.30	7.27 ± 3.84	0.179
Neutrophilic granulocyte percentage	248.91 ± 105.51	286.46 ± 109.46	<0.001	252.74 ± 107.78	274.40 ± 114.88	0.032	216.81 ± 108.32	247.69 ± 110.54	0.002
Percentage of lymphocytes	26.95 ± 118.66	18.65 ± 9.84	<0.001	21.75 ± 9.43	21.20 ± 17.72	0.049	23.80 ± 10.91	20.16 ± 9.29	<0.001
Absolute eosinophil count	15.12 ± 16.50	12.59 ± 15.68	0.001	14.09 ± 12.82	13.52 ± 15.98	0.087	17.08 ± 16.77	17.32 ± 18.99	0.365
Plateletcrit	0.19 ± 0.09	0.19 ± 0.06	0.516	0.97 ± 11.78	0.19 ± 0.06	0.44	0.25 ± 0.86	0.19 ± 0.06	0.524
Red blood cell distribution width	13.48 ± 1.45	13.43 ± 1.35	0.967	13.57 ± 1.61	13.27 ± 1.53	0.136	13.71 ± 7.08	13.36 ± 1.19	0.377
Mean platelet volume	10.48 ± 5.61	10.11 ± 1.26	0.734	10.25 ± 1.30	9.92 ± 1.17	0.024	10.61 ± 1.31	10.42 ± 1.25	0.101
Platelet Distribution Width	16.05 ± 1.52	15.91 ± 1.34	0.639	16.16 ± 1.17	15.93 ± 1.23	0.008	12.93 ± 2.97	12.97 ± 2.83	0.472
C-Reactive Protein	187.51 ± 172.49	210.22 ± 176.06	0.022	205.12 ± 168.28	196.15 ± 175.59	0.565	325.33 ± 208.07	332.22 ± 211.67	0.736
Arterial Oxygen Partial Pressure	89.26 ± 22.12	87.04 ± 21.31	0.019	87.39 ± 18.84	90.20 ± 25.21	0.947	84.18 ± 9.92	82.86 ± 15.81	0.053
Arterial Carbon Dioxide Partial Pressure	38.85 ± 3.80	41.32 ± 5.86	<0.001	39.05 ± 3.61	42.13 ± 7.69	<0.001	40.13 ± 5.69	58.63 ± 277.77	<0.001
Gender			0.008			0.119			0.778
Female	80 (14.44)	78 (21.37)		38 (16.67)	39 (23.49)		19 (9.18)	22 (8.03)	
Male	474 (85.56)	287 (78.63)		190 (83.33)	127 (76.51)		188 (90.82)	252 (91.97)	
Smoke			0.956			0.292			1
No	189 (34.12)	126 (34.52)		75 (32.89)	64 (38.55)		89 (43.00)	118 (43.07)	
Yes	365 (65.88)	239 (65.48)		153 (67.11)	102 (61.45)		118 (57.00)	156 (56.93)	

Univariate and multivariate analyses revealed age, gender, and arterial partial pressure of carbon dioxide (PaCO2) as predictors in the clinical model; these variables also independently predicted the severity of chronic obstructive pulmonary disease (COPD), as detailed in Table 2. The area under the receiver operating characteristic curve (AUC) values for the clinical model were 0.634 (95% confidence interval: 0.596–0.671) in the training set, 0.63 (95% confidence interval: 0.572–0.688) in the internal validation set, and 0.616 (95% confidence interval: 0.566–0.666) in the external validation set, as presented in Table 3.

**Table 2:** Univariable and multivariable analysis of clinical features.

Variable	Univariable analysis		Multivariable analysis	
	OR [95%CI]	*p* value	OR [95%CI]	*p* value
Plateletcrit	0.128 [0.073, 0.224]	<0.01	0.399 [0.067, 2.373]	0.397
Gender	0.606 [0.535, 0.685]	<0.01	0.485 [0.34, 0.693]	0.001
Smoke	0.655 [0.571, 0.751]	<0.01	1.032 [0.77, 1.383]	0.858
Triglyceride	0.699 [0.638, 0.765]	<0.01	0.887 [0.732, 1.075]	0.305
Mean platelet volume	0.96 [0.949, 0.97]	<0.01	0.97 [0.924, 1.017]	0.288
White blood cell count	0.961 [0.947, 0.975]	<0.01	1.025 [0.987, 1.065]	0.277
Red blood cell distribution width	0.969 [0.962, 0.977]	<0.01	0.964 [0.891, 1.043]	0.442
Platelet distribution Width	0.974 [0.968, 0.98]	<0.01	0.925 [0.856, 1.001]	0.102
Percentage of lymphocytes	0.977 [0.972, 0.982]	<0.01	0.971 [0.94, 1.002]	0.12
Absolute eosinophil count	0.981 [0.975, 0.986]	<0.01	0.996 [0.987, 1.005]	0.484
Albumin	0.989 [0.986, 0.992]	<0.01	0.996 [0.982, 1.01]	0.661
Arterial carbon dioxide partial pressure	0.991 [0.988, 0.994]	<0.01	1.15 [1.115, 1.185]	0
Age	0.994 [0.993, 0.996]	<0.01	0.981 [0.967, 0.995]	0.028
Arterial oxygen partial pressure	0.995 [0.994, 0.996]	<0.01	0.994 [0.989, 1]	0.111
Alkaline Phosphatase	0.996 [0.994, 0.997]	<0.01	0.998 [0.995, 1.001]	0.228
Globulin	0.996 [0.995, 0.997]	<0.01	0.997 [0.994, 1]	0.105
BMI	0.999 [0.999, 0.999]	<0.01	0.999 [0.999, 1]	0.067
C-reactive protein	0.999 [0.999, 1]	<0.01	1.001 [1, 1.002]	0.056
Neutrophilic granulocyte percentage	0.999 [0.999, 0.999]	<0.01	1 [0.997, 1.004]	0.818

**Table 3:** Performance of four models across different cohorts.

Model	AUC	Accuracy	95% CI	Sensitivity	Specificity	PPV	NPV	Precision	Recall	F1	Threshold	Cohort
Clinic	0.634	0.656	0.5963–0.6708	0.471	0.778	0.583	0.691	0.583	0.471	0.521	0.402	train
Rad	0.76	0.712	0.7274–0.7918	0.652	0.751	0.633	0.766	0.633	0.652	0.642	0.374	train
DTL	0.805	0.737	0.7775–0.8333	0.69	0.767	0.661	0.79	0.661	0.69	0.676	0.468	train
DLR	0.822	0.743	0.7940–0.8491	0.775	0.722	0.648	0.83	0.648	0.775	0.706	0.379	train
Combined	0.839	0.777	0.8134–0.8652	0.679	0.841	0.738	0.799	0.738	0.679	0.708	0.493	train
Clinic	0.63	0.637	0.5720–0.6875	0.506	0.732	0.579	0.671	0.579	0.506	0.54	0.39	val
Rad	0.729	0.69	0.6783–0.7792	0.627	0.737	0.634	0.73	0.634	0.627	0.63	0.396	val
DTL	0.757	0.68	0.7088–0.8043	0.711	0.658	0.602	0.758	0.602	0.711	0.652	0.391	val
DLR	0.74	0.688	0.6912–0.7894	0.681	0.693	0.617	0.749	0.617	0.681	0.648	0.386	val
Combined	0.759	0.706	0.7100–0.8074	0.735	0.684	0.629	0.78	0.629	0.735	0.678	0.326	val
Clinic	0.616	0.593	0.5663–0.6659	0.467	0.758	0.719	0.518	0.719	0.467	0.566	0.398	test
Rad	0.704	0.674	0.6571–0.7506	0.701	0.638	0.719	0.617	0.719	0.701	0.71	0.356	test
DTL	0.752	0.711	0.7091–0.7957	0.785	0.614	0.729	0.683	0.729	0.785	0.756	0.38	test
DLR	0.759	0.699	0.7169–0.8021	0.664	0.744	0.774	0.626	0.774	0.664	0.715	0.39	test
Combined	0.767	0.701	0.7247–0.8097	0.646	0.773	0.79	0.623	0.79	0.646	0.711	0.382	test

The models include the Clinic (clinical model signature), Rad (radiomics signature), DTL (deep learning signature), DLR (deep learning radiomics signature), and Combined (a combination of clinical, deep learning, and radiomics signatures).

### Feature selection and model construction

**Figure 3:**
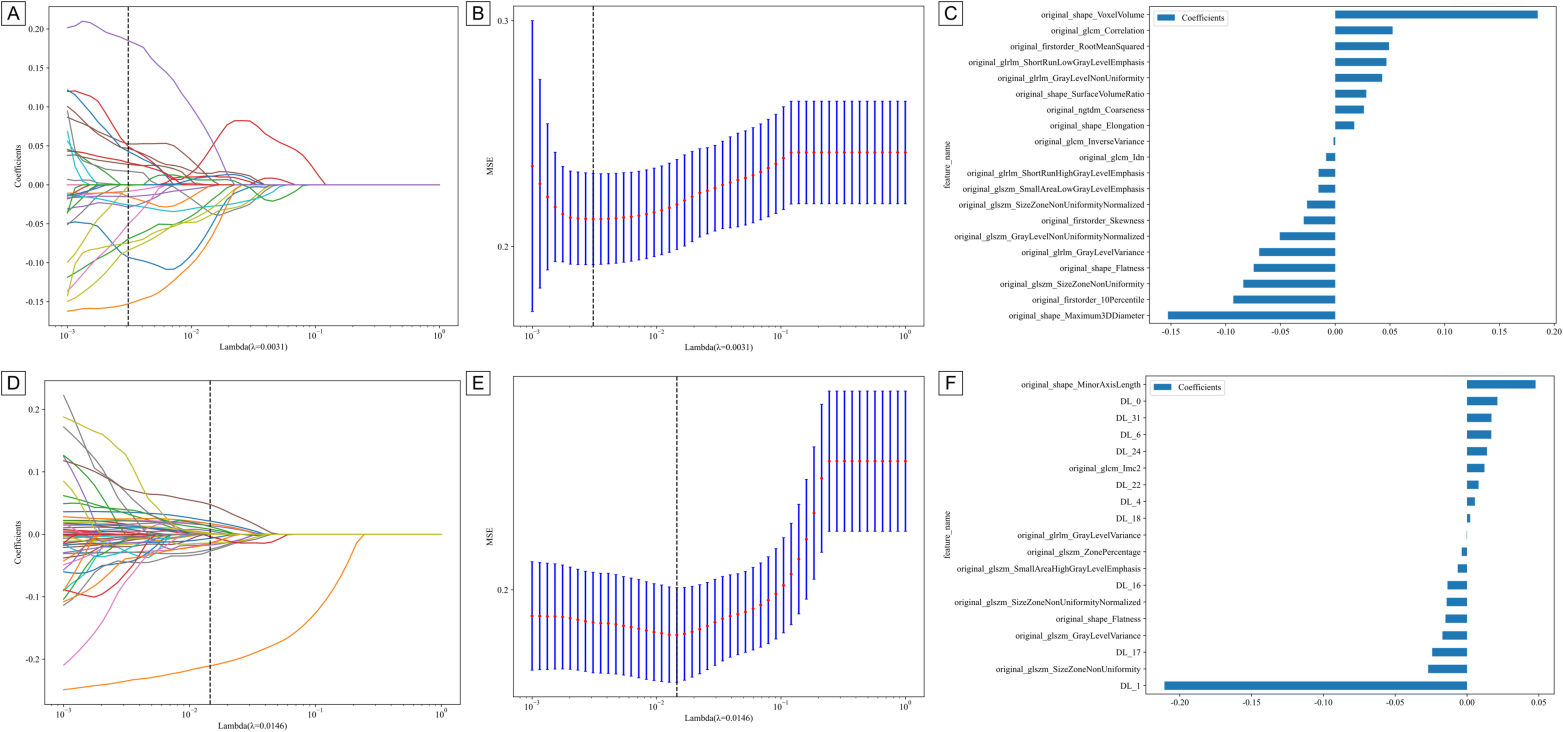
**(A,D)** Represent the LASSO for radiomics and DLR features. **(B,E)** Represent the MSE for radiomics and DLR features. **(C,F)** Represent the feature weights for radiomics and DLR features.

Radiological features and three-dimensional deep learning features were extracted from images based on the automatic segmentation of chest CT scans. Reproducibility was assessed using the intraclass correlation coefficient (ICC). Following t-tests, Spearman correlation analyses, and LASSO screening, 20 optimal radiomic features with non-zero coefficients were selected, and a radiological model was constructed. The coefficients and average standard errors derived from five-fold cross-validation are presented in Figures 3A,B, while Figure 3C shows the values of the selected features with non-zero coefficients. The specific formula for screening radiomic features is detailed in Supplementary Data 3.

**Figure 4:**
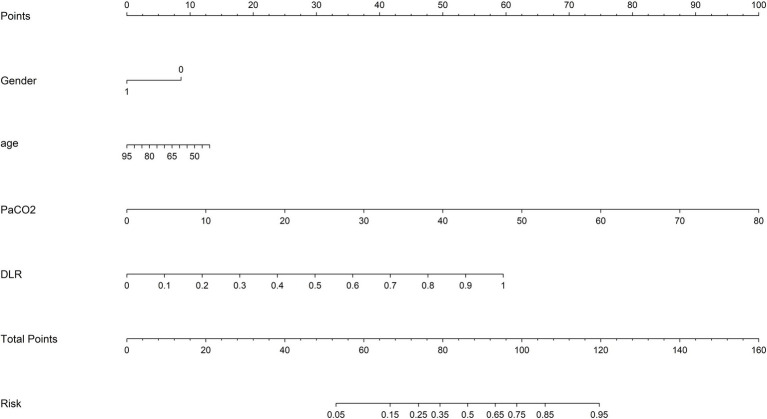
The clinical application of nomogram in the prediction of COPD grades.

The ResNet50 architecture, a convolutional neural network (CNN), was employed to extract deep learning features, yielding a total of 2048 features. After compression, 32 deep learning features were retained to construct the deep learning model (DTL). To enhance the accuracy of predicting chronic obstructive pulmonary disease (COPD) severity, radiological and deep learning features were integrated. Through t-tests, Spearman correlation coefficients, and LASSO regression analysis, 19 features with non-zero coefficients were selected (Figures 3D–F), enabling the construction of a deep learning radiological model (DLR). The formula used to screen DLR features is provided in Supplementary Data 4. Finally, age, gender, and partial pressure of carbon dioxide (PaCO2) were combined with the DLR model, and a nomogram model was constructed using multivariate logistic regression. Figure 4 presents a nomogram designed specifically for clinical use.

The construction of the nomogram (Figure 4) is based on the regression coefficients of the final multivariate logistic regression model. The ‘Points’ for each predictor variable is calculated using the following formula: For continuous variables (age, PaCO₂ and DLR-score), multiply the regression coefficient corresponding to the variable value by the value of the variable (or the standardized value), then scale it proportionally to the integer scale of 0–100; for categorical variables (gender), assign a fixed score directly based on the regression coefficient. Specifically, the score contributed by each variable = (the regression coefficient of the variable / the sum of the absolute values of the regression coefficients of all variables) × 100. The total score of each variable is obtained by adding them up, and then the corresponding ‘probability of severe COPD’ is read from the bottom by mapping it through the logistic regression function. Users can find the score corresponding to each variable above the graph 4 based on the patient’s age, gender, PaCO₂and DLR-score, sum them up, and read the corresponding prediction probability at the bottom. The combined model of this study can be deployed as an automated calculation tool or transformed into a manual nomogram.

### Comparison of clinical models, imaging-based models, deep learning models, DLR models and combined models

**Figure 5:**
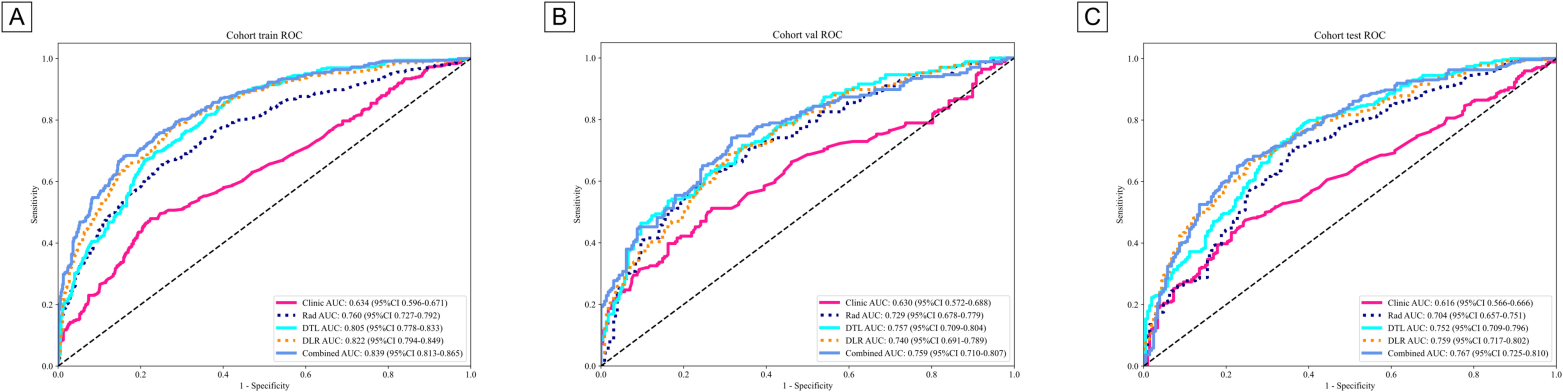
ROC curve of different models in the **(A)** train, **(B)** internal validation, and **(C)** external validation cohort, respectively.

**Figure 6:**
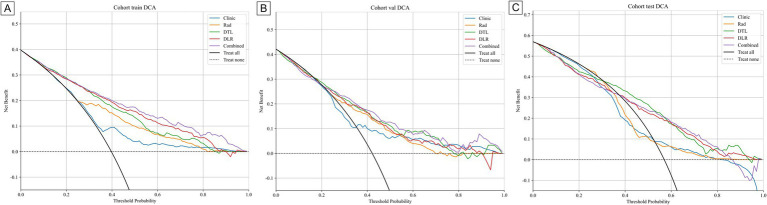
DCA curve of different models in the **(A)** train, **(B)** internal validation, and **(C)** external validation cohort, respectively.

**Figure 7:**
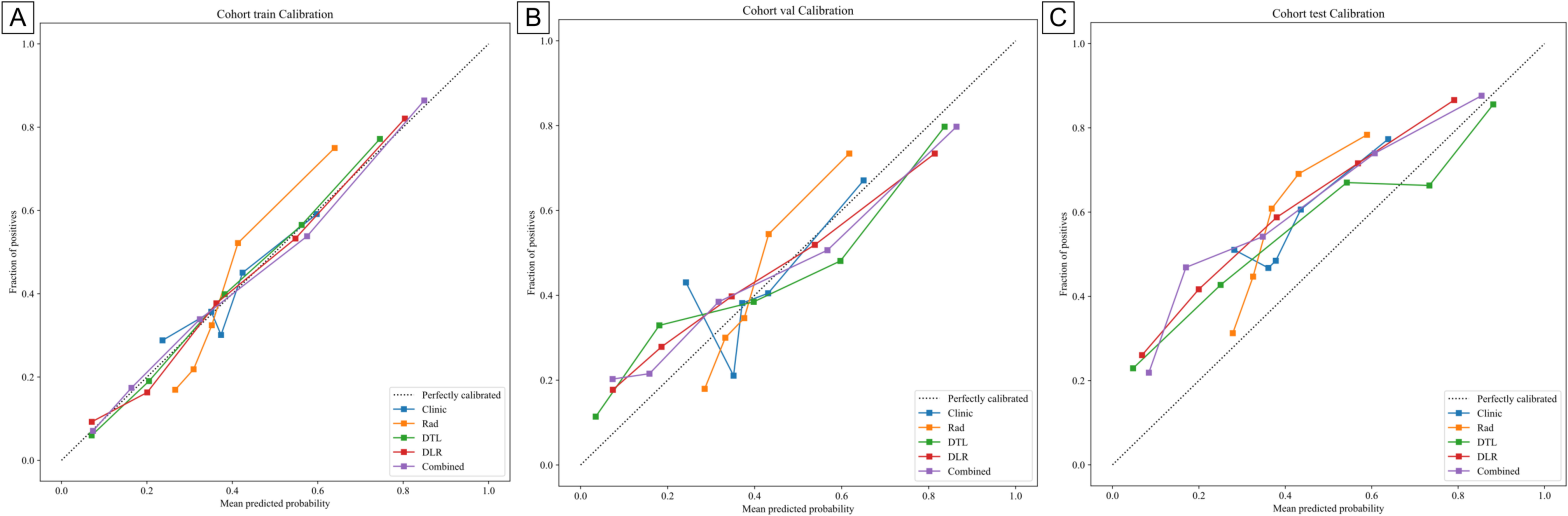
Calibration curve of different models in the **(A)** train, **(B)** internal validation, and **(C)** external validation cohort, respectively.

**Figure 8:**
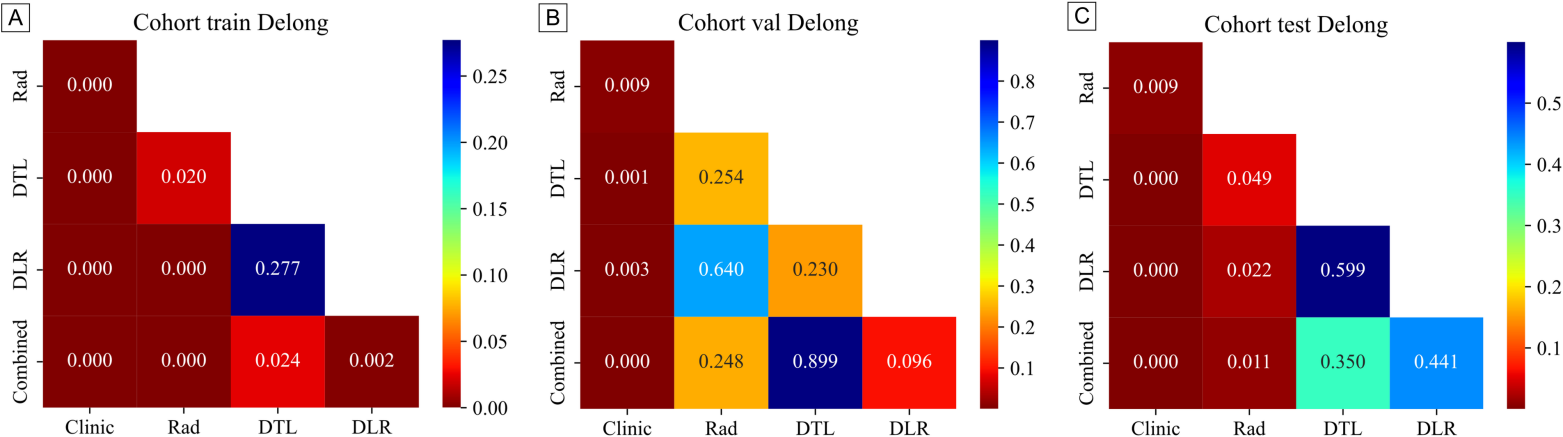
Presents the DeLong test results for different models evaluated in **(A)** the training cohort, **(B)** the internal validation cohort, and **(C)** the external validation cohort.

Table 3 presents the performance of clinical, radiomics, deep learning (DTL), deep learning radiomics (DLR), and combined models. In the training cohort (Figure 5A), the combined model achieved the highest area under the curve (AUC) of 0.839, followed by the DLR model (0.822), the DTL model (0.805), and the radiomics (Rad) model (0.76). The clinical model had a relatively low AUC of 0.634. In the internal validation cohort (Figure 5B), the combined model again outperformed others with an AUC of 0.759, followed by the DTL model (0.757), the DLR model (0.74), and the Rad model (0.729). The clinical model had an AUC of 0.63. In the external validation cohort (Figure 5C), the combined model had the highest AUC (0.767), followed by the DLR model (0.759), the DTL model (0.752), and the Rad model (0.704). The clinical model had an AUC of 0.616. These results consistently demonstrate the low performance of the clinical model across cohorts, highlighting its limitations for standalone use. The Rad and DTL models showed moderate performance, with one slightly outperforming the other in different contexts. The combined model generally exhibited the best AUC performance. Although there were differences in the collection methods, our combined model still achieved a stable AUC (0.767) in the external validation, indicating that it has certain robustness to multi-center heterogeneity. These findings underscore the importance of advanced modeling techniques and multimodal data fusion in predicting chronic obstructive pulmonary disease (COPD) staging. Figure 6 displays decision curve analysis (DCA) results, which were used to assess the clinical applicability of the models. The combined model demonstrated a higher net benefit compared to the others, indicating a superior benefit–risk ratio in clinical decision-making. Figure 7 presents calibration curves for each cohort, showing the agreement between model-predicted probabilities and observed outcomes. Additionally, Figure 8 shows the results of discriminative performance comparisons among the models using the DeLong test. The combined model, the clinical model, and the radiomics model all showed significant differences across all cohorts (*p* ≤ 0.05), demonstrating that multimodal fusion (clinical + radiomics + deep learning) has significantly improved performance compared to single-modal or dual-modal models. Based on the analysis, the combined nomogram model exhibited superior predictive efficacy, performing well in terms of both discrimination and calibration (Table 4).

**Table 4:** The *p*-values of the Hosmer-Lemeshow test for all the cohort.

Clinic	Rad	DTL	DLR	Combined	Cohort
0.04	0.002	0.007	0.485	0.026	train
0.003	0.025	0.235	0.182	0.19	val
0.211	0.016	0.027	0.537	0.38	test

## Discussion

We developed and validated a composite nomogram that integrates full-lung radiomics features derived from chest CT scans, deep learning features, and clinical independent predictors to assess COPD severity. Radiomics and deep learning features independently stratify COPD severity, demonstrating that chest CT can evaluate both lung structure and functional status.

Mild COPD patients are often overlooked due to asymptomatic or mild symptoms (19, 20), whereas severe COPD significantly impairs quality of life and escalates treatment costs (21). Differentiated treatment strategies based on COPD severity are critical for patient prognosis (9), necessitating an early, rapid, and accessible diagnostic method for staging. Prior studies have identified morphological CT changes—such as bronchial wall thickening, tracheal shape alterations, and total lung emphysema percentage—as correlates of severe COPD (22). Machine learning advancements have enabled novel staging approaches: Chen TT (23) and Rueda R (24) combined machine learning models with clinical and biochemical markers, while Gu YF et al. developed a COPD severity prediction model using biochemical and immunological parameters, though its 62.7% accuracy limited clinical utility (25). Given COPD’s heterogeneous lung involvement, leveraging CT to detect abnormal textures and assess disease status is crucial (26, 27). Sui H et al. improved severity classification by integrating lung parenchyma shape, size, distribution, and airway morphology into a machine learning framework (28).

However, selecting and capturing relevant images from large datasets remains challenging despite machine learning advancements. Radiomics enables high-throughput extraction of quantitative features from medical images, with Li Z (29) and Zhou T (30) demonstrating its efficacy in COPD severity classification. Some researchers refined grading by analyzing radiomics features across individual lung lobes, capturing localized CT characteristics more effectively than whole-lung approaches (31). These studies underscore radiomics’ feasibility for COPD staging. With artificial intelligence progress, deep learning has gained traction: Sara Rezvanjou et al. combined 2D tMPR images and 3D lung views to classify COPD using airway data (32), while Zou X et al. developed a cost-effective but less sensitive deep learning model for COPD staging using chest X-rays and clinical parameters (33). Our study integrates chest CT images, clinical parameters, radiomics, and deep learning features into a clinically applicable nomogram, enhancing CT’s functional assessment value.

Our model extracts texture, edge, structural, and shape information from CT images, yielding richer feature descriptions for disease prediction. Adding deep learning features significantly improved diagnostic performance compared to models using only clinical or radiomics features. The final nomogram incorporates clinical factors (age, gender, PaCO2), radiomics features, and deep learning features. Decision curve analysis (DCA) confirmed its superior net benefit over clinical models across diagnostic thresholds, supporting its clinical utility. By improving COPD severity classification accuracy, our nomogram aids in managing acute exacerbations, personalizing treatment interventions, controlling symptoms, and slowing disease progression.

This study has limitations. First, as a retrospective analysis from three institutions, the third center’s smaller sample size may limit generalizability; a prospective multicenter study is planned to optimize the cohort. At the same time, explore coordination algorithms such as ComBat to further enhance the generalization ability of features. Second, variables with >30% missing values were excluded, warranting closer attention in future research. Third, the single-timepoint design did not assess longitudinal lung changes in the same patients. Future directions include integrating traditional CT features and quantitative parameters to enhance model efficacy and using delta radiomics to study COPD dynamics. Fourthly, the binary outcome definition adopted in this study means that this model cannot replace the detailed GOLD four-stage assessment. Future research should increase the sample size, especially by including more patients in GOLD stage I and GOLD stage IV, and use ordinal logistic regression or machine learning multi-classification methods to achieve four-category prediction.

## Conclusion

This study utilized automatic segmentation technology to extract the entire lung parenchyma from CT images. By integrating clinical characteristics of independent risk factors, whole-lung radiomic features, and deep learning features, a novel combined nomogram was constructed to identify COPD severity. The study also demonstrated the added value of chest CT in evaluating lung structure and functional status.

## Data Availability

The original contributions presented in the study are included in the article/Supplementary material, further inquiries can be directed to the corresponding author.

## Footnotes

1
https://github.com/OnekeyAI-Platform/onekey

2
www.itksnap.org

3
http://pyradiomics.readthedocs.io
