# Supplementary material for: Development and validation of a multimodal clinical-radiomics-deep learning nomogram based on automated chest CT segmentation for classifying COPD severity: a multicenter study
Source: Front Med (Lausanne). 2026 May 13;13:1831103. doi: 10.3389/fmed.2026.1831103 (PMC13212252; doi:10.3389/fmed.2026.1831103)
Supplement: Supplementary file 1 [file Data_Sheet_1.DOCX]

Supplementary Data

# Supplementary Data1

CT acquisition protocols for different scanners

| **Scanning equipment** | **Center 1** | | | **Center 2** | | | **Center 3** |
| --- | --- | --- | --- | --- | --- | --- | --- |
|  | Somatom Definition AS | Somatom Perspective | GE Optima540 | Brilliance CT 16 | uCT 530 | Somatom Force | Brilliance CT 16 |
| Tube voltage (kV) | 120 | 120 | 120 | 120 | 120 | 120 | 120 |
| Tube current (mAs) | 40 | 40 | 40 | 40 | 30 | 40 | 40 |
| Pitch | 0.758 | 0.758 | 0.758 | 0.758 | 0.758 | 0.758 | 0.758 |
| Collimation (mm) | 0.6×64 | 0.6×64 | 0.6 × 16 | 0.6 × 16 | 0.6 × 40 | 0.6 × 256 | 0.6 × 16 |
| Slice thickness (mm) | 1 | 1 | 0.6/1 | 0.6/1 | 1 | 1 | 0.6/1 |
| Matrix | 512 × 512 | 512 × 512 | 512 × 512 | 512 × 512 | 512 × 512 | 512 × 512 | 512 × 512 |

# Supplementary Data 2

# Algorithm Workflow:

(1).Data Preprocessing: The algorithm begins by loading the CT scan data and converting it into a format suitable for processing. This involves resampling the images to standardize the spatial resolution, ensuring that the subsequent analysis is consistent regardless of the original scan resolution.

(2).Initial Binarization: The next step involves transforming the CT image into a binary mask. This mask distinguishes between potential lung tissue and other structures based on intensity thresholds, effectively isolating areas that are likely to represent lungs or air-filled spaces.

(3).Refinement of the Binary Mask: The binary mask is then refined through a series of operations:

Connected Component Analysis: The algorithm identifies and labels distinct regions in the mask. This helps in distinguishing between different anatomical structures, such as lungs, bronchi, and external air.

Exclusion of Non-Lung Areas: The algorithm systematically excludes regions that are unlikely to be part of the lungs, such as areas connected to the corners of the image or regions with volumes that are too small or too large to represent lung tissue.

Morphological Refinement: The mask is further refined by filling small holes within the lung regions and separating the left and right lungs if they are connected.

(4).Final Segmentation: After the refinement process, the algorithm produces two separate masks for the left and right lungs. These masks are combined into a final output that accurately represents the lung regions within the 3D CT scan.

(5).Output Generation: The final step involves converting the segmented lung masks back into an image format and saving them for further use. This allows the segmented lungs to be utilized in various downstream applications, such as disease detection, volumetric analysis, or 3D visualization.

**Supplementary Data 3:**

Rad_score = 0.39717083786724716

-0.093051 * original_firstorder_10Percentile

+0.049130 * original_firstorder_RootMeanSquared

-0.028771 * original_firstorder_Skewness

+0.052429 * original_glcm_Correlation

-0.008346 * original_glcm_Idn

-0.001562 * original_glcm_InverseVariance

+0.042868 * original_glrlm_GrayLevelNonUniformity

-0.069427 * original_glrlm_GrayLevelVariance

-0.015200 * original_glrlm_ShortRunHighGrayLevelEmphasis

+0.046791 * original_glrlm_ShortRunLowGrayLevelEmphasis

-0.050651 * original_glszm_GrayLevelNonUniformityNormalized

-0.083942 * original_glszm_SizeZoneNonUniformity

-0.025715 * original_glszm_SizeZoneNonUniformityNormalized

-0.015308 * original_glszm_SmallAreaLowGrayLevelEmphasis

+0.026206 * original_ngtdm_Coarseness

+0.017360 * original_shape_Elongation

-0.074483 * original_shape_Flatness

-0.152742 * original_shape_Maximum3DDiameter

+0.028363 * original_shape_SurfaceVolumeRatio

+0.184913 * original_shape_VoxelVolume

**Supplementary Data 4:**

Rad_score= 0.39717083786724705

+0.021156 * DL_0

-0.210671 * DL_1

+0.005507 * DL_4

+0.016886 * DL_6

-0.013595 * DL_16

-0.024219 * DL_17

+0.002186 * DL_18

+0.008046 * DL_22

+0.013918 * DL_24

+0.017023 * DL_31

+0.012151 * original_glcm_Imc2

-0.000254 * original_glrlm_GrayLevelVariance

-0.017118 * original_glszm_GrayLevelVariance

-0.027054 * original_glszm_SizeZoneNonUniformity

-0.014177 * original_glszm_SizeZoneNonUniformityNormalized

-0.006484 * original_glszm_SmallAreaHighGrayLevelEmphasis

-0.003694 * original_glszm_ZonePercentage

-0.014997 * original_shape_Flatness

+0.047728 * original_shape_MinorAxisLength

**Supplementary Data 5:**

**The standard units of each clinical indicator:**

BMI:kg/m²

CPR:mg/L

Albumin:g/L

Globulin:g/L

Triglyceride:mmol/L

Alkaline Phosphatase:IU/L

White blood cell count：×10⁹/L

Absolute eosinophil count：×10⁹/L

Plateletcrit：%

Red blood cell distribution width：%

Mean platelet volume：fl

Platelet Distribution Width（PDW）:%

Erythrocyte Sedimentation Rate（ESR）:mm/h

Procalcitonin(PCT）：μg/L

Arterial Oxygen Partial Pressure（PaO2):mmHg

Arterial Carbon Dioxide Partial Pressure（PaCO2):mmHg
